# A Brief Update on Inter-lab Quality Control (ILQC) Activities for COVID-19 Reverse Transcription-Polymerase Chain Reaction (RT-PCR) by All India Institute of Medical Sciences (AIIMS) Nagpur, India

**DOI:** 10.7759/cureus.59645

**Published:** 2024-05-04

**Authors:** Vishal Shete, Meena Mishra, Neeta Gade, Soumyabrata Nag, Pooja Shendre

**Affiliations:** 1 Microbiology, All India Institute of Medical Sciences, Nagpur, Nagpur, IND; 2 Microbiology, Government Medical College, Nagpur, Nagpur, IND

**Keywords:** rt-pcr covid-19, quality control, sars-cov-2, inter-lab quality control, ilqc

## Abstract

Inter-lab quality control (ILQC) is vital for ensuring reliable test results, especially when laboratories are using assays authorized for newly emerging pathogens. The Indian Council of Medical Research (ICMR), New Delhi, had developed a network of laboratories to assess the quality of real-time reverse transcription (RT) polymerase chain reaction (PCR) assays used in India to detect severe acute respiratory syndrome coronavirus 2 (SARS-CoV-2). In a three-tier ILQC lab structure, All India Institute of Medical Sciences (AIIMS) Nagpur, an institute of national importance & a tertiary care hospital, was designated as a state quality control (QC) lab for the region of Maharashtra. ILQC activities were planned biannually. The ICMR had assigned 22 government and 19 private SARS-CoV-2 RT-PCR testing laboratories, under the Department of Microbiology, AIIMS Nagpur. AIIMS Nagpur had conducted four ILQC activities during 2020-2021. The finding of the ILQC assessment (cumulative includes all four ILQC) conducted by AIIMS Nagpur revealed that the results of 77% of laboratories were 100% concordant, the results of 14% of laboratories were 90%, and very few laboratories (i.e. 9%) showed <90% concordant.

## Editorial

India has expanded the COVID-19 laboratory with different testing platforms in a phased manner. Over five months, the number of laboratories within the country rose from 14 in February 2020 to more than 1,596 in August 2020 [1]. Herculean efforts were undertaken to set up reverse transcription-polymerase chain reaction (RT-PCR) laboratories all across the country to tackle the pandemic. Rolling out a novel test amid the pandemic in resource-constraint settings where the majority of the laboratories lack accreditation significantly heightens the risk of errors [2]. The quality management system within laboratories is crucial to ensure accurate and reliable results and reduce the risk of errors. To have reliable results, the inter-lab quality control (ILQC) program for COVID-19 was initiated by the ICMR, New Delhi, on 5 April 2020. The objective of ILQC activities was to assess the quality of functioning of laboratories performing RT-PCR for COVID-19.

ILQC of the labs was implemented through a three-tier structure, viz., national QC laboratories, state QC laboratories, and testing laboratories. The national quality control lab (i.e. ICMR-National Institute of Virology (NIV)) conducted ILQC activities for state QC labs, whereas state QC labs conducted ILQC activities for other laboratories in their respective catchment areas. ILQC activities was planned biannually. To begin with, 33 laboratories had been given the status of state quality control lab for COVID-19. Among these, the All India Institute of Medical Sciences (AIIMS) Nagpur was one of the state quality control labs conducting the QC activity of laboratories in Maharashtra.

**Figure 1:**
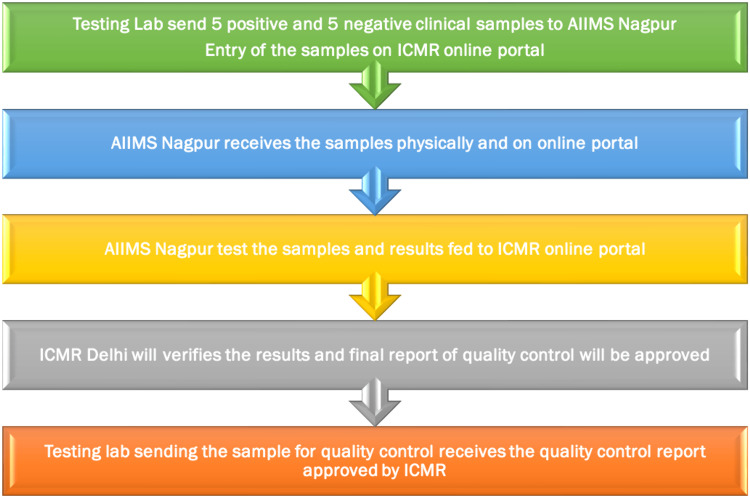
Flowchart of the methodology

To facilitate the whole process, the ICMR launched an online portal for ILQC activities [3]. Five positive clinical samples (nasopharyngeal and pharyngeal swabs in a viral transport medium) and five negative clinical samples were randomly selected over one week and had to be sent by the testing lab to AIIMS Nagpur. Positive samples with CT values between 25 and 35 were preferred. The testing lab had to make all entries (five positive and five negative samples) into the quality control portal after sending the samples to AIIMS Nagpur. After testing the samples, AIIMS Nagpur fed the results onto ILQC portals [3]. As a part of the ICMR protocol, the samples received for the ILQC were processed using the ICMR-NIV multiplex single-tube SARS-CoV-2 assay. Samples are encoded to obscure their identity from the QC lab. AIIMS Nagpur processed the samples within one week of receiving them, after which the results were entered into the ILQC portal. Both the testing lab and the QC lab refrained from sharing results. Then the results matching and verification were done by ICMR, New Delhi, and were displayed on the ICMR portal (Figure 1).

The ICMR-NIV multiplex single-tube SARS-CoV-2 assay includes three genes E gene, ORF 1ab, RdRP of SARS-CoV-2 genes, and one housekeeping gene B actin. The assay had one screening and two confirmatory viral genomic region targets, which reduced the risk of false negatives.

Department of Microbiology, AIIMS Nagpur, conducted four ILQCs activities. In 2020, the ILQC activity frequency was quarterly and carried out in the third and fourth quarters ( i.e. in August 2020 and October 2020, respectively). In 2021, the frequency of ILQC activity was changed to biannually: in January 2021 and July 2021. Seventeen laboratories participated in the first ILQC activity, 30 in the second ILQC activity, 29 in the third ILQC activity, and 34 in the fourth ILQC activity. The concordance of the ILQC samples varies from 60% to 100%. During the latest ILQC activity conducted in July 2021, 26 labs showed 100% concordance, five labs showed 90% concordance, and three labs showed <90% concordance (Table 1).

Although the ILQC program ensures quality with the different testing platforms, multiple factors hinder the results. Different RT-PCR kits with different primers and probes and the target sequences can affect the assay performance [4]. Laboratory practice standards and personnel skills in the relevant technical and safety procedures affect the RT-PCR assay results [4]. Additionally, the presence of amplification inhibitors in the sample or insufficient organisms in the sample arise from inappropriate collection, transportation, or handling [4]. Sample storage conditions and irregularity in cold chain transport affect the assay results. This includes elevating cycle threshold values in nucleic acid tests, potentially causing positive cases and leading to misclassifying the positive cases as negative [5].

To conclude, ILQC activities played an important role during the COVID pandemic and have contributed towards the indirect assessment of RT-PCR testing as a whole, comprising various components such as proper collection and transport of samples, the skill of the lab personnel, and quality of the kits and reagents. Laboratories with open-system RTPCR for COVID-19 used different kits with different target genes. Consequently, ensuring the result's accuracy and reliability remains a constant concern. Moreover, the few laboratories where the results were discordant indicated the appropriate corrective action to achieve better results in the next ILQC activities.
